# Giant suppression of phononic heat transport in a quantum magnet BiCu_2_PO_6_

**DOI:** 10.1038/srep36970

**Published:** 2016-11-15

**Authors:** Byung-Gu Jeon, B. Koteswararao, C. B. Park, G. J. Shu, S. C. Riggs, E. G. Moon, S. B. Chung, F. C. Chou, Kee Hoon Kim

**Affiliations:** 1 CeNSCMR, Department of Physics and Astronomy, Seoul National University, Seoul 151-747, Korea; 2 Center of Condensed Matter Sciences, National Taiwan University, Taipei 10617, Taiwan; 3 National High Magnetic Field Laboratory, Florida State University, Tallahassee, Florida 32310, USA; 4 Department of Physics, Korea Advanced Institute of Science and Technology, Daejeon 305-701, Korea; 5 Center for Correlated Electron Systems, Institute for Basic Science (IBS), Seoul 151-747, Korea; 6 Department of Physics and Astronomy, Seoul National University, Seoul 151-747, Korea; 7 Institute of Applied Physics, Seoul National University, Seoul 151-747, Korea

## Abstract

Thermal transport of quantum magnets has elucidated the nature of low energy elementary excitations and complex interplay between those excited states via strong scattering of thermal carriers. BiCu_2_PO_6_ is a unique frustrated spin-ladder compound exhibiting highly anisotropic spin excitations that contain both itinerant and localized dispersion characters along the *b*- and *a*-axes respectively. Here, we investigate thermal conductivity *κ* of BiCu_2_PO_6_ under high magnetic fields (*H*) of up to 30 tesla. A dip-feature in *κ*, located at ~15 K at zero-*H* along all crystallographic directions, moves gradually toward lower temperature (*T*) with increasing *H*, thus resulting in giant suppression by a factor of ~30 near the critical magnetic field of *H*_c_ ≅ 23.5 tesla. The giant *H-* and *T-*dependent suppression of *κ* can be explained by the combined result of resonant scattering of phononic heat carriers with magnetic energy levels and increased phonon scattering due to enhanced spin fluctuation at *H*_c_, unequivocally revealing the existence of strong spin-phonon coupling. Moreover, we find an experimental indication that the remaining magnetic heat transport along the *b*-axis becomes almost gapless at the magnetic quantum critical point realized at *H*_c_.

Thermal transport of quasi low-dimensional (D) quantum spin systems has been widely investigated to uncover both the intriguing quantum mechanical nature of magnetism and the low dimensionality of spin networks. For instance, anisotropic thermal conductivity (*κ*) of quantum magnets along the chain, ladder leg or in-plane directions clearly reflects the kinetics of magnetic quasiparticles that is highly direction dependent^1^,^2^. Similar to other thermodynamic properties, *κ* also reflects low-energy quasiparticle excitations of the quantum magnets. The metallic power-law behavior observed in the temperature (*T*)-dependent thermal conductivity, *κ(T*), proximity to zero temperature indicates gapless spinon excitation in 1D spin chains^3^ or spinon Fermi surfaces in 2D quantum spin liquids^4^.

The magnetic field (*H*)-dependent behavior of *κ*, *κ(H*), further provides fruitful information on the evolution of spin excitations and their coupling with the lattice degree of freedom. *H* dependence of *κ* arises for various reasons, such as extra heat conduction due to magnetic quasiparticles or additional scattering of phononic heat carriers via strong spin-phonon coupling^5^,^6^,^7^,^8^,^9^,^10^,^11^. It is thus expected that the *H* dependence will be most pronounced near the magnetic quantum critical point (QCP) where the spin gap is closed to excite magnetic quasiparticles easily or spin fluctuation becomes maximized. In this respect, the investigations of magneto-thermal conductivity near the critical magnetic field (*H*_c_) for a quantum phase transition can be a promising way to understand the spin-phonon interaction as well as the magnetism itself.

BiCu_2_PO_6_ is a new kind of spin-ladder compound that forms a unique spin ground state of a spin dimer crystal or valence bond solid. Its crystal structure inherently contains geometric spin frustration within the ladder (Fig. 1a); it is composed of distorted *S* = 1/2 (Cu^2+^ ions) spin chains with antiferromagnetic (AF) interactions *J*_leg_ (≈138 K) and *J*_NNN_ (≈73 K) along the *b* axis, thereby having frustrated spin interactions. These chains are coupled to one another along the *c* axis through another AF interaction *J*_rung_ (≈58 K) to form a two-leg spin ladder in the *bc* plane^12^,^13^,^14^. Various thermodynamic, light scattering and nuclear magnetic resonance measurements have indicated anisotropic spin-gap with the excitation energy (Δ/*k*_B_) ranging from 32 to 45 K, without any long-range spin order down to 0.1 K^12^,^13^,^14^,^15^,^16^,^17^,^18^,^19^,^20^. The application of magnetic field then leads to reduction in the spin gap (Fig. 1b) and induces cascades of magnetic phase transitions above critical magnetic fields *μ*_0_*H*_c_ = 20–24 T, the values of which depend on the direction of the applied field^21^,^22^.

Inelastic neutron scattering studies on BiCu_2_PO_6_ have also uncovered intriguing spin excitations distinct from other conventional spin ladders. The spin excitation branches, 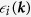 (*i* = 0, +1, −1), become split even at *H* = 0 because of giant anisotropic spin interactions originating from strong spin-orbit coupling, which can be described by antisymmetric Dzyaloshinskii-Moriya (D-M) and symmetric anisotropic spin exchange terms. Thus, the ground state can no longer be described as singlet only and the excited states exhibit the characteristics of mixed singlet and triplet states. It has thus resulted that spin excitations become highly dispersive particularly along the b* axis and almost dispersionless along the a* axis^19^. Naturally, the minima of the three excitation branches at *H* = 0 becomes different, giving rise to the three excitation gaps, Δ_0_/*k*_B_ ≈ 19 K, Δ_+1_/*k*_B_ ≈ 36 K and Δ_−1_/*k*_B_ ≈ 45 K satisfying Δ_0_ < Δ_+1_ < Δ_−1_ (Fig. 1b)^20^. In the magnetic Raman spectra, in particular, the polarization dependency in the singlet bound modes has been observed, which constitutes a strong evidence of two-triplon bound states with the binding energy of approximately 33 K^18^. Furthermore, the observation of softening in the singlet modes suggested the melting of a spin dimer crystal (or valence bond solid) through the singlet fluctuations^18^. It has been also suggested that the triplons can even become fractionalized and reconstructed into a soliton lattice at higher magnetic fields above *H*_c_^17^.

All those unusual spin excitation spectra and postulated high field phases suggest that study of thermal transport could be useful to understand the evolution of spin and phonon excitations of BiCu_2_PO_6_ in the high field region where conventional neutron scattering cannot be performed. In particular, it would be interesting to study how the geometrical frustration and strong D-M interaction can manifest themselves in the magnetic and phononic heat transport across the magnetic QCP. Indeed, a recent thermal transport study of BiCu_2_PO_6_ in a low-*H* region has found a pronounced suppression of *κ* at ~15 K, resulting in a double-peak feature in *κ(T*) along all crystallographic directions^23^. This behavior is highly unusual, as the double-peak feature has been found only along the leg direction in most other spin ladder compounds because of the separate contributions of phononic and magnetic heat carriers^1^,^2^. To shed light on the origin of this anomalous behavior, the application of high *H* across *H*_c_ should be also useful because it can provide information on the *H*-dependent variation of the double-peak feature and the intriguing nature of the strong spin-phonon coupling.

Here, we report an investigation of thermal conductivity in BiCu_2_PO_6_ single crystals under high magnetic fields up to 30 T. We found that the dip located between the double peaks in *κ(T*) moves systematically toward lower temperatures as *H* is increased to *H*_c_, which result in the strongest field-induced suppression of *κ* known among the magnetic materials (by a factor of ~30). Such large *T-* and *H-*dependent variations in *κ* could be explained in terms of the resonant scattering of the phonons induced by magnetic transitions involving two-triplon bound states^8^,^18^. By developing a theoretical model for phononic *κ* having resonant scatterings with the three spin excitation branches, we could extract information on the evolution of those magnetic energy levels across *H*_c_. Furthermore, *κ*_*b*_(*T*) measured along the ladder leg direction revealed remnant magnetic contribution that scaled almost linearly in temperature at *H*_c_., supporting that the magnetic heat transport nearly becomes gapless at the magnetic QCP.

## Results

### Zero-field thermal conductivity of BiCu_2_PO_6_

**Figure 1:**
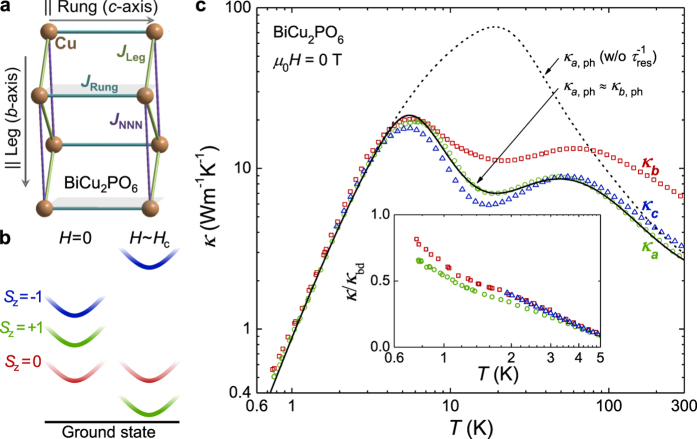
Crystal structure, magnetic energy levels and zero-field thermal conductivity of BiCu_2_PO_6_. (**a**) The schematic crystal structure of the Cu^2+^ *S* = 1/2 frustrated two-leg ladder in BiCu_2_PO_6_. (**b**) Expected magnetic energy levels of BiCu_2_PO_6_ at *H* = 0 and *H* ~ *H*_c_. Minima of the excitation branches satisfy Δ_0_ ≈ 19 K < Δ_+1_ ≈ 36 K < Δ_−1_ ≈ 45 K at *H* = 0 20. (**c**) Temperature-dependent thermal conductivity of BiCu_2_PO_6_ single crystals at 0 T and along each direction (*κ*_*a*_, *κ*_*b*_ and *κ*_*c*_). The solid black line represents the calculated phonon component along the *a* axis (*κ*_*a*,ph_), and dashed black line represents *κ*_*a*,ph_ without resonant scattering processes. The inset shows the measured data divided by the boundary scattering limit of the phonon thermal conductivity (*κ/κ*_bd_).

Figure 1c shows the *κ*_*i*_(*T*) (*i* = *a*, *b*, *c*) of the BiCu_2_PO_6_ single crystals at *μ*_0_*H* = 0 T. Note that above 10 K, *κ*_*b*_ becomes clearly higher than *κ*_*a*_ and *κ*_*c*_, thereby implying that the heat conduction along the leg (*b*) direction is higher than that along the interlayer (*a*) or rung (*c*) direction. The anisotropy observed in Fig. 1c does not appear to originate from the anisotropic phonon thermal conductivity (*κ*_ph_). To estimate the intrinsic anisotropy in *κ*_ph_, we rescaled *κ* in the low-temperature regime with respect to *κ*_bd_*, i.e., κ* in the ‘boundary scattering limit’. From the kinetic theory, the boundary scattering limit of phononic thermal conductivity can be expressed as *κ*_bd_ = *c*_ph_*v*_ph_*l*_bd_/3, where *c*_ph_ is a phononic specific heat of a crystal approximately given as *βT*^3^, *v*_ph_ is an averaged sound velocity determined from the Debye temperature *θ*_D_ = *v(ħ*/*k*_B_)(6*π*^2^*n*/*V*)^1/3^ = 330 K 24 (*n*/*V*: number density of atoms), and *l*_bd_ is the boundary scattering limit of the phonon mean free path determined from the sample geometry as 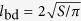 (*S*: cross section of the sample)^25^. The calculated *κ*/*κ*_bd_ values under the assumption of isotropic phonon dispersion thus reflect the intrinsic anisotropy in the phonon transport, independent of the crystal geometry. The *κ*/*κ*_bd_ curves in the inset of Fig. 1c exhibit anisotropy of less than 20%, which is not sufficient to explain the anisotropy in *κ* above 10 K, which shows a difference of more than 200%. Different strengths of umklapp scattering can also be ruled out as an explanation because it would lead to different peak positions in *κ*_ph_(*T*), which is inconsistent with the results shown in Fig. 1c 26. We therefore argue that the *κ*_ph_ along the *a* axis (*κ*_*a*,ph_) should be similar to the *κ*_ph_ along the *b* axis (*κ*_*b,*ph_), *i.e*., *κ*_*a,*ph_ ≈ *κ*_*b,*ph_ in BiCu_2_PO_6_, regardless of its crystallographic structure. Thus, the extra heat transport along the leg (*b*) direction could be magnetic in its origin, which indeed reflects the nature of anisotropic magnetic excitation of BiCu_2_PO_6_.

The most conspicuous feature in the shapes of *κ*_*i*_(*T*), as shown in Fig. 1c, is the presence of a dip feature sandwiched by the double peak structure, which is observed for every crystallographic direction. Such a double-peak structure has been commonly observed in quasi low-dimensional magnets (spin chains or spin ladders), particularly in only one direction where the magnetic thermal transport reaches a maximum^1^,^2^. It is therefore anomalous to observe the double peaks along every direction in one of spin ladder compounds, BiCu_2_PO_6_. Moreover, none of the peaks in *κ(T*) appears to be either a solely phononic or a solely magnetic peak; because the magnetic excitation along the reciprocal *a* axis is known to be non-dispersive^19^ and the velocity of magnetic quasiparticles (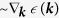) along the *a* axis should be much smaller than that along any other direction. Therefore, it is unlikely that magnetic thermal conductivity *κ*_mag_ can produce a separate peak along the *a* axis. In addition, attempts to estimate *κ*_ph_ in every direction from the single peak only below 7 K (or above 20 K) resulted in extremely small *κ*_ph_ at high (low) temperatures. Therefore, it is necessary to consider another mechanism for explaining the double peak feature in *κ*_ph_ along every direction.

### Giant suppression of thermal conductivity under magnetic field

**Figure 2:**
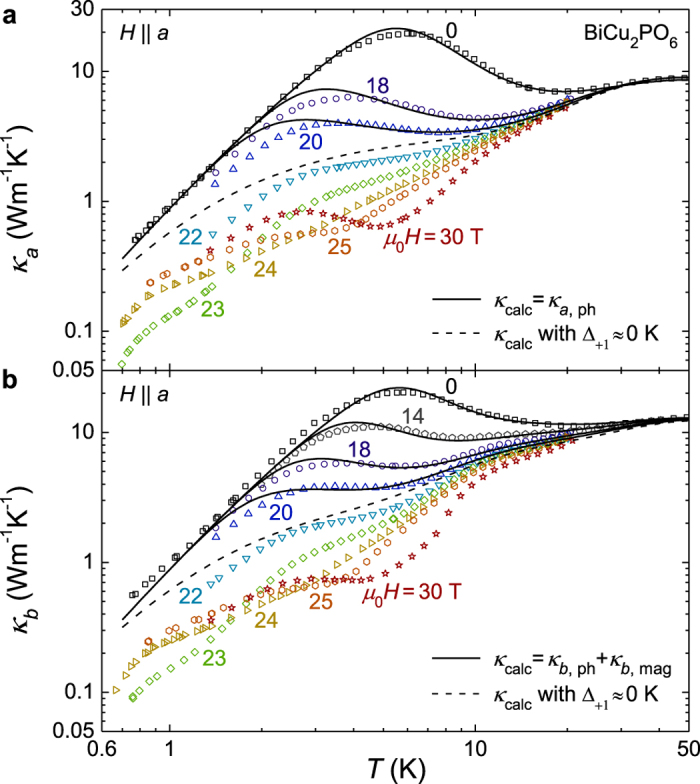
High-field thermal conductivity of BiCu_2_PO_6_. Thermal conductivity of BiCu_2_PO_6_ single crystals under magnetic fields of up to 30 T is plotted (symbols), including a comparison with the calculated thermal conductivity from the transport theory for fields of up to 20 T (solid lines). In each case the thermal gradient was applied either along the (**a**) *a* or (**b**) *b* axis. The dashed lines represent the thermal conductivity along the *a* and *b* axes with a minimum Δ_+1_(*H*) ≈ 0, which represents the lowest limit on the thermal conductivity estimated from the resonant scattering model.

**Figure 3:**
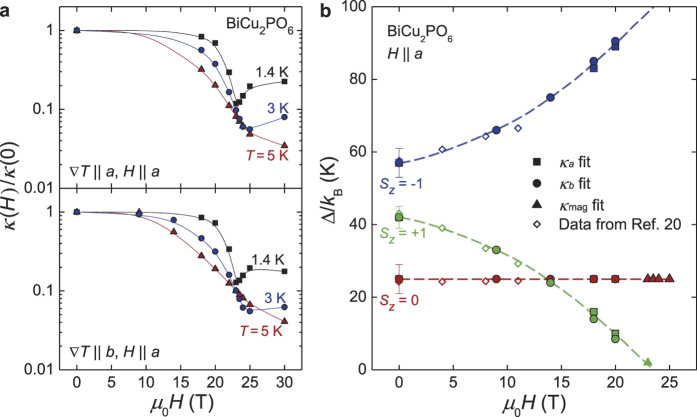
Magnetic field dependence of the thermal conductivity (*κ(H*)/*κ*(0)) and the magnetic excitation gaps. (**a)** The magneto-thermal conductivity values at 1.4 K, 3 K and 5 K are extracted from *κ(T*) curves at fixed magnetic fields. The solid lines are guides for the eye. **(b)** Magnetic field dependence of the magnetic excitation gaps for fields of up to 25 T. The values were estimated from the analyses of the phonon and magnetic thermal conductivities (solid symbols). The minimum spin excitation energies determined from the inelastic neutron scattering results have been multiplied by a factor of 1.25 (small open symbols)^20^. The error bars represent typical uncertainties of the values estimated from the phonon thermal conductivity analysis. The dashed lines are guides for the eye.

To better understand the origin of the double-peak feature, we performed *κ(H*) measurements along the *a* and *b* axes for fields of up to *μ*_0_*H* = 30 T (Fig. 2). As *H* is approached to *H*_c_, the dip features in both directions systematically shift toward lower temperatures, resulting in very small *κ* at low temperatures. Above *H*_c_ where the spin-ordered phases appear, *κ* at low temperatures around 1.4 K is again enhanced. Figure 3a shows the magneto-thermal conductivity (*κ(H*)/*κ*(0)) at *T* = 1.4, 3 and 5 K, as summarized from the results in Fig. 2. At 1.4 K, it is clearly observed that *κ(H*)/*κ*(0) sharply decreases near *H*_c_^22^. However, as temperature is increased, the decreasing region becomes widened and gradually shifts toward higher *H*. It is noteworthy that *κ* is suppressed by a factor of ~30 at *T* = 5 K and *μ*_0_*H* = 30 T, which is, to our knowledge, the largest magneto-thermal conductivity observed in magnetic materials. For example, SrCu_2_(BO_3_)_2_, Ba_3_Mn_2_O_8_, and hexagonal HoMnO_3_ showed similar field-induced *κ* suppression by a factor of ~4 at *μ*_0_*H* = 17 T 8, ~10 at 14 T 6, and ~9 at ~3 T 9, respectively. Therefore, the observation of such a large *κ* suppression under magnetic fields is a unique case. In comparison, such large suppression of *κ* has been also observed in the hexagonal HoMnO_3_ as a function of temperature near the Ho^3+^ spin ordering as compared with YMnO_3_. This has been attributed to the strong spin-phonon coupling and magnetic fluctuations arising from the spin frustration^27^.

### Resonant scattering of phonon by magnetic excitations in BiCu_2_PO_6_

To understand the anomalous *κ(T*) behavior and strong *H* dependence of BiCu_2_PO_6_ in a quantitative level, we consider here the model of the resonant scattering of phonons by magnetic excitations, which has been formerly applied in SrCu_2_(BO_3_)_2_ to explain a similar dip feature^8^. SrCu_2_(BO_3_)_2_ represents an archetypal, exactly-solvable spin-frustrated networks, in which its ground state can be described as a bound two-dimer system having two *S* = 1/2 spins in each dimer^28^. Similarly, the frustrated spin interactions along the leg direction in BiCu_2_PO_6_ may provide a similar binding effect between adjacent dimers formed by the rung coupling as evidenced by a recent Raman scattering study^18^.

In such a bound two-dimer system, a phonon-assisted magnetic transition between the excited states with the same *S*_*z*_ value, *i.e.*, one of the thermally excited *S* = 1 one-triplet, one-singlet states and *S* = 1 bound triplet states, having energy difference of Δ_s_ can be induced, accompanied by absorbing or emitting a phonon of energy *ћω* ≈ Δ_s_ due to the spin-phonon coupling. The entire two-step process, magnetic excitation and de-excitation, that restores the initial spin state can then be regarded as *ω*-dependent elastic scattering of the phonons, and the phonon relaxation rate *τ*^−1^ will be directly proportional to the transition rate of the magnetic excitation. In the isolated two-dimer system, there is no correlation between the momentum directions of the phonon at initial and final states. Although the original resonant scattering model^8^ assumed a flat dispersion of the magnetic excitation spectra, it is likely applicable to any spin-gapped system with dispersive excitations because most excitations appear within a narrow ***k*** region near the band minima. Furthermore, the evolution of the spin excitation gaps under *H* is likely to modify the transition rate, resulting in the strength change of the resonant phonon scattering (Fig. 1b).

Thermal transport by phonons or magnetic quasiparticles can be generally described by


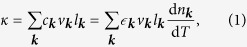


where *c*_***k***_, *v*_***k***_, *l*_***k***_ and *n*_***k***_ represent the heat capacity, velocity, mean free path and statistical occupation of quasiparticles of wavevector ***k***. Applying the Debye approximation to Eq. (1) leads to the Callaway model for *κ*_ph_, the temperature dependence of which is predominantly determined by the phonon relaxation rate 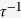 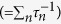
26,29.

To calculate the relaxation rate by the resonant scattering (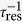), we considered three nondegenerate excitation branches (*S*_z_ = 0, +1, −1), which are split even at zero field by the anisotropic spin interactions, and are subject to change under *H* by the Zeeman effect^20^. The phonon scattering rate due to such excited levels can be described as follows (Methods):


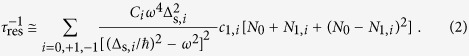


Here, *C*_*i*_ is the overall factor related to the strength of the spin-phonon coupling, 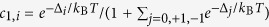 is the statistical occupation of thermally excited states with *S*_z_  =  *i* (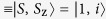), Δ_*i*_ is the *H*-dependent energy gap between the ground state (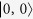) and an excited state (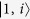), and Δ_s,*i*_ is the *H*-independent resonance energy determined by the energy difference between the magnetic excited states of the two-dimer system. In SrCu_2_(BO_3_)_2_ where degenerate triplet states are realized, Δ_s_ = Δ − *B*, where *B* is the binding energy of the two triplons. In addition, 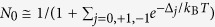 and 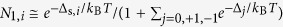 are the probabilities of the excitation and de-excitation processes, respectively.

The overall factors *C*_*i*_ are here assumed as magnetic field-independent parameters as long as *H* does not exceed *H*_c_^8^. Although there is a possibility that strength of the elastic spin-phonon coupling decreases as the field-induced spin polarization appears even below *H*_c_ due to the D-M interactions and paramagnetic contributions^14^, its magnetic moment is sufficiently small (less than 0.005 *μ*_B_/Cu^2+^ below *H*_c_^21^) and thus the effect could be considered as higher-order terms.

### Quantitative analyses of thermal transport with magnetic energy levels

The double-peak feature observed in the *κ* data (Fig. 1c) can be explained in terms of the resonant scattering model for *κ*_ph_ described in Eqs (1) and (2), with the numerical parameters listed in Table 1. Because the dispersion of the magnetic excitation is nearly flat along the *a* axis^19^, we apply the resonant scattering model to *κ*_*a*_. At *μ*_0_*H* = 0 T, the dip at ~15 K can be quantitatively explained as a consequence of the resonant scattering in *κ*_ph_; the calculated *κ* (solid line in Fig. 1c) shows good agreement with the experimental *κ*_*a*_ in the entire temperature range. In contrast, the predicted curve for *κ*_ph_ without considering the resonant scattering (dashed line in Fig. 1c) exhibits a typical single-peak feature expected in nonmagnetic, insulating materials^26^. This observation supports the hypothesis that the resonant scattering in *κ*_ph_ is the origin of the double peaks and the dip observed in Fig. 1c.

One of intriguing findings obtained from the best fit is that the overall constant related to the strength of the resonant scattering for *S*_*z*_ = 0 branch (*C*_0_) is at least one order of magnitude smaller than the constant for *S*_*z*_ = 1 (*C*_1_) or *S*_*z*_ = −1 (*C*_−1_) branches (Table 1). This indicates that the resonant scattering from the transition between *S* = 0 one-triplet, one-singlet and *S* = 0 bound triplet could be negligible as compared with other magnetic excitations, which may constitute a clue for developing further microscopic theories on the resonant scattering.

To understand the *κ* data under magnetic fields (Fig. 2), we apply the same resonant scattering model, assuming that the energy gaps are dependent on *H* (see Fig. 1b and Table 2). In a recent inelastic neutron scattering study^20^, each magnetic level exhibited the Zeeman splitting up to *μ*_0_*H* = 11 T such that the excitation gaps could be roughly described as follows: Δ_0_(*H*) = Δ_0_(0), Δ_+1_(*H*) = Δ_+1_(0) − Δ(*H*) and Δ_−1_(*H*) = Δ_−1_(0) + Δ(*H*). Here, Δ(*H*) is an *H*-dependent parameter. The *κ*_*a*_ curves calculated for different Δ(*H*) (solid lines in Fig. 2a) are in good agreement with the experimental data, particularly for temperatures above 5 K and fields of up to 20 T. This finding clearly indicates that the double-peak feature in *κ*_*a*_(*T*) and the suppression of the peak at low temperatures can be mostly explained by *κ*_ph_ based on the resonant scattering model.

The filled squares in Fig. 3b summarize the *H* dependence of the three energy gaps, Δ_*i*_(*H*), for fields of up to 20 T, as obtained from the best fits to the results presented in Fig. 2a and Table 2. For example, the energy gaps of the three excitation branches at *μ*_0_*H* = 0 T were found to be Δ_0_/*k*_B_ ≈ 25 K, Δ_+1_/*k*_B_ ≈ 42 K and Δ_−1_/*k*_B_ ≈ 57 K. All the *H*-dependent energy gaps Δ_*i*_(*H*) obtained by fitting *κ*_*a*_(*T*) (filled squares) are qualitatively consistent with the recent neutron scattering results (open symbols), although they are larger by ~25% than those obtained from the minimum energies of the magnetic excitation spectra^20^. Because magnetic excitations involving phonon scattering are not confined only to the minima of these dispersive branches, it seems reasonable that the effective gap values estimated from the *κ*_ph_ fits are higher.

Based on the three excitation gaps obtained from our fit (see Table 2), the averaged gap value becomes Δ~41 K. As the resonance energy (Δ_s_) obtained from our fit was approximately 32 K, the triplon binding energy *B* is then roughly estimated to be *B* = Δ − Δ_s_ ≈ 9 K. Recent Raman scattering result^18^ pointed that the binding energy of the bound singlet is 2*B* ≈ 33 K and thus *B* ≈ 16 K, which is rather close to the *B* from our fits although it is a rough estimation based on the averaged spin gap value. Such agreement supports the presence of the triplon bound state in the resonant phonon scattering processes, and justifies the application of the above model into the BiCu_2_PO_6_ system.

Along the *b* axis, however, the experimental *κ*_*b*_ data cannot be explained solely by the resonant scattering model in *κ*_ph_. Figure 1c shows that the experimental *κ*_*b*_ data are overall larger than *κ*_*a,*ph_ (≈*κ*_*b,*ph_). To better fit the experimental *κ*_*b*_ data, an additional contribution from *κ*_mag_ was indeed essential. This seems consistent with the fact that the inelastic neutron scattering studies found most dispersive spin excitation branches along the reciprocal b* axis^19^. To calculate *κ*_mag_ explicitly, we have extended Eq. (1) to derive new formula for *κ*_mag_ when the excited triplet states are nondegenerate (Methods). The result is


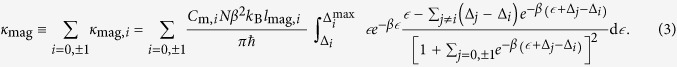


Here, *β* = 1/*k*_B_*T*, and *l*_mag,*i*_ is the magnetic mean free path of the *i*-th branch. The solid lines in Fig. 2b show the calculated *κ*_*b*_ ( = *κ*_*b*,ph_ + *κ*_*b*,mag_) results for fields of up to *μ*_0_*H* = 20 T, in which *κ*_*b*,ph_ is assumed to be the same as *κ*_*a*,ph_. As a result of including the additional *κ*_mag_, we could achieve good agreement between the experimental *κ*_*b*_ data and the calculated ones, particularly at temperatures above 5 K and fields of up to *μ*_0_*H* = 20 T. The excitation gap values obtained from the fit are also plotted in Fig. 3b as filled circles, which overall show good agreement with the excitation gaps obtained from the fit along the *a* axis.

However, when 20 T < *H* < *H*_c_, the theoretically predicted values of both *κ*_*a*_ (=*κ*_*a*,ph_) and *κ*_*b*_ (=*κ*_*b*,ph_ + *κ*_*b*,mag_) clearly overestimated the experimental data in a wide temperature window below 10 K. For example, the experimental data at *μ*_0_*H* = 22 T are far more suppressed below ~10 K than the theoretically predicted *κ*_*a*_ and *κ*_*b*_, even after applying a minimum Δ_+1_(*H*) ≈ 0, which yields the most significant suppression of *κ*_ph_ in the resonant scattering model (dashed lines in Fig. 2). This observation directly points out that the additional scattering of heat carriers, beyond the resonant scattering model adopted here, should exist particularly near *H*_c_. Two possible origins could be postulated. First, when the spin energy gap becomes sufficiently small, the resonant scattering model, which assumes flat dispersion, may not fully account for the effect of dispersion in the magnetic energy levels. However, it is unlikely that the dispersion effect will bring up enhanced resonant scattering at *H* ≈ *H*_c_. More importantly, beyond the resonant scattering, the additional nonresonant scattering of phononic heat carriers due to the spin fluctuation seems to play a role because the spin fluctuation most likely increases at the magnetic quantum critical point.

### Field dependence of magnetic thermal conductivity near the critical magnetic field

**Figure 4:**
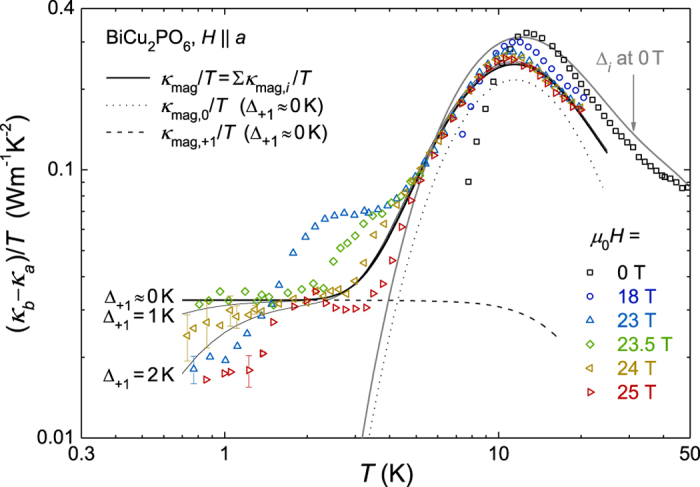
High-field magnetic thermal conductivity divided by temperature, as estimated from the difference between the *b* axis and *a* axis components. The thick solid lines are theoretical curves calculated based on the transport theory with Δ_0_/*k*_B_ ≈ 25 K, Δ_+1_/*k*_B_ ≈ 0 K and Δ_−1_/*k*_B_ ≈ 99 K (*H* = *H*_c_, black) and Δ_0_/*k*_B_ ≈ 25 K, Δ_+1_/*k*_B_ ≈ 42 K and Δ_−1_/*k*_B_ ≈ 57 K (Δ_*i*_ at 0 T, gray). The calculated magnetic components at *H* = *H*_c_ from the *S*_*z*_ = 0 (dotted line) and *S*_*z*_ = +1 (dashed line) branches are individually plotted. The calculated magnetic thermal conductivity curves with finite spin gaps (Δ_+1_/*k*_B_ = 1 K and 2 K) are also included (thin solid lines).

The very large suppression of *κ*_ph_ at *H* ≤ *H*_c_ in both directions suggests the possibility that *κ*_*b,*mag_ can be extracted approximately at low temperatures and at *H* ≈ *H*_c_ where *κ*_ph_ becomes sufficiently small due to the enhanced phonon scattering^30^. Assuming that the relation *κ*_*a,*ph_ ≈ *κ*_*b,*ph_ is valid at *T* > 7 K or *H* ≈ *H*_c_, we subtracted the *κ*_*a*_ from the *κ*_*b*_ data to plot (*κ*_*b*_ − *κ*_*a*_)/*T*, as shown in Fig. 4. The (*κ*_*b*_ − *κ*_*a*_)/*T* at *T* > 7 K, *μ*_0_*H* = 0 T shows a clear peak at *T*~13 K, which is a typical feature of *κ*_mag_ in the gapped spin ladder systems^1^,^30^. The *κ*_mag_ peak is almost insensitive to the magnetic field, suggesting that Δ_0_ is indeed magnetic field-independent and magnetic mean free path is not strongly subject to the resonant phonon scattering. Interestingly, the most constant behavior is observed at *μ*_0_*H*_c_ = 23.5 T in the low-temperature limit down to 0.8 K, whereas the neighboring data at 23 T or 24 T show clear decreases. This observation implies that the magnetic thermal conductivity becomes nearly linear in the low-temperature limit at the critical field *μ*_0_*H*_c_ = 23.5 T.

However, it was difficult to analyze the experimental data at *μ*_0_*H* ≤ 22 T and ~1 K < *T* < 7 K in a more quantitative level because anomalous peak- or dip-like structures were superimposed on top of the exponential tail of *κ*_mag_ (*e.g.* gray solid line in Fig. 4). Although the anisotropy ratio between *κ*_*a*,ph_ and *κ*_*b,*ph_ (=*κ*_*a*,ph_/*κ*_*b,*ph_) is expected to be less than 20%, the anisotropy of *κ*_ph_ that is presumably related to the sample geometry and/or directional dependence of the spin-phonon coupling could be still non-negligible to produce such additional peak- or dip-like structures in the (*κ*_*b*_ − *κ*_*a*_)/*T* data.

With the caveat of having such additional structures, to understand whether *κ*_mag_ can still explain the low temperature experimental data of the (*κ*_*b*_ − *κ*_*a*_)/*T* near *H* ≈ *H*_c_, we calculated *κ*_mag,*i*_/*T (i* = 0, +1, −1) individually with fitted parameters listed in Tables 1 and 3 and plotted *κ*_mag_/*T* ≡ ∑_*i*_ _=_ _0,±1_*κ*_mag,*i*_/*T*, *κ*_mag,0_/*T* and *κ*_mag,+1_/*T* in Fig. 4. Here, the energy gaps were assumed to be Δ_0_/*k*_B_ ≈ 25 K, Δ_+1_/*k*_B_ ≈ 0 K and Δ_−1_/*k*_B_ ≈ 99 K. The calculated *κ*_mag_/*T* results well reproduced the overall shape of (*κ*_*b*_ − *κ*_*a*_)/*T* at *μ*_0_*H* = 0 T and 23.5 T, including the magnetic field-independent broad peak at 11 K~13 K. [The hump at ~3 K may be attributable to the anisotropic phonon component that does not exactly cancel out in the (*κ*_*b*_ − *κ*_*a*_)/*T* plot.] Moreover, the results for *κ*_mag,+1_/*T* with Δ_+1_/*k*_B_ ≈ 0 K (at *H* = *H*_c_) can explain the constant behavior observed in the low-temperature limit, whereas the *κ*_mag,−1_/*T* data contribute only very little. This finding indicates that a gapped magnetic excitation to the *S*_z_ = 0 branch and a nearly-gapless magnetic excitation to *S*_z_ = +1 coexist at *H* = *H*_c_. When a finite energy gap of Δ_+1_/*k*_B_ = 1 K or 2 K is used, the calculated *κ*_mag_/*T* results decrease at low temperatures, showing a clear deviation from the constant *κ*_mag_/*T* behavior. Therefore, the energy gap Δ_+1_/*k*_B_ at *μ*_0_*H*_c_ = 23.5 T, if any, could be less than 1 K to reproduce constant *κ*_mag_/*T* behavior for temperatures down to 0.8 K. Moreover, the energy gap Δ_+1_/*k*_B_ at 23 T is expected to be approximately 2 K. Based on the experimental data in this work, we estimate that the excitation gaps are close to Δ_0_/*k*_B_ ≈ 25 K, Δ_+1_/*k*_B_ ≈ 0 K and Δ_−1_/*k*_B_ ≈ 99 K at *H* = *H*_c_. Triangles in Fig. 3b and Table 2 summarize those estimated gap values around *H*_c_. The results in Fig. 3b exhibiting the continuous evolution of the three excited levels corroborates that a magnetic quantum phase transition indeed occurs at *H* = *H*_c_ due to the crossing of the energy levels between the *S*_z_ = +1 excited state and the magnetic ground state.

Almost negligible energy gap in the *S*_z_ = +1 branch around *H*_c_ expected from the *T* dependence of the magnetic thermal conductivity is rather peculiar, because the spin gap in the spin-dimer systems with strong D-M interaction is not expected to be closed completely even at *H*_c_ due to the mixing of singlet and triplet states. It may indicate that opening of the spin gap by the anisotropic D-M interaction is comparable or less than the energy scale of ~0.8 K^17^, or the D-M interaction is strong enough so that it cannot be treated as a higher-order effect and different physics might be need to understand our observation. Investigations of the critical behavior of BiCu_2_PO_6_ at lower temperatures and related theoretical works will be necessary for understanding its magnetism around the critical field.

## Discussion

One fundamentally interesting question is why the phonon transport in BiCu_2_PO_6_ is subject to strong resonant and nonresonant scatterings with the magnetic energy levels and then result in giant magneto-thermal conductivity. A common feature in SrCu_2_(BO_3_)_2_ and BiCu_2_PO_6_ where the resonant scattering process becomes conspicuous, is the existence of spin frustration and anisotropic spin interaction described by the Dzyaloshinskii-Moriya interaction^20^,^31^. Hence, the spin-orbit coupling represented by the Dzyaloshinskii-Moriya interaction in the frustrated spin network might be one of necessary conditions for having the dominant resonant phonon scattering. Moreover, the conventional phonon scattering due to spin fluctuation effect, beyond the level described by the resonant scattering model, seems to be also significant particularly near *H*_c_ and at low temperatures in BiCu_2_PO_6_. It gives rise to the additional suppression of *κ* by a factor of ~3 while the resonant scattering leads to the *κ* suppression typically by a factor of ~10 according to our fit results. Similar kinds of large *κ* suppression have been observed in multiferroics with strong magnetoelectric (or spin-phonon) coupling and attributed to the enhanced spin-phonon scattering near the magnetic phase transitions^9^,^10^,^11^.

Related to this, it is noteworthy that the spin dimer (valence bond solid) state in BiCu_2_PO_6_ lies in the distorted, non-coplanar network of CuO_4_ squares that share their edges and corners along the rung and leg directions of the ladders, respectively. Hence, both spin couplings along the rung and leg directions should be greatly affected by the Cu-O-Cu superexchange interaction, which is inherently sensitive to the local Cu-O-Cu boding angle. This structural characteristic seems to provide one natural route to favor strong spin-phonon coupling in BiCu_2_PO_6_. It is further noteworthy that the field-induced electric polarization and magnetoelectric effect have been recently found in the magnetically-ordered, high-field phases of BiCu_2_PO_6_, clearly supporting the existence of strong spin-phonon coupling (B.-G. Jeon *et al.*, unpublished data). Therefore, the presence of both resonant and nonresonant phonon-scattering processes via the strong spin-orbit and spin-phonon coupling in frustrated spin networks seems to be the salient feature for causing the giant suppression of *κ* near the magnetic quantum phase transition in BiCu_2_PO_6_.

In summary, we have observed the unprecedentedly large suppression of the phononic thermal conductivity in the vicinity of magnetic quantum critical point in BiCu_2_PO_6_ single crystals. In contrast to other quasi-low-dimensional magnets, in which both phononic and magnetic heat conduction exist almost independently, both resonant scattering of phononic heat carriers with the magnetic excitation levels and the additional phonon scattering due to the magnetic fluctuation were found to be dominant sources of the *κ* suppression. We claim the importance of spin-orbit and spin-phonon coupling in the frustrated spin network to boost up such resonant and nonresonant scatterings of phononic heat carriers with the spin degree of freedom. Finally, our work also points to an interesting possibility of using the quantum magnet as a field-tunable thermal insulator under high magnetic field environment while superconducting materials have often been used as a thermal insulator at low field regions below their upper critical fields.

## Methods

### Sample preparation

High-quality single crystalline BiCu_2_PO_6_ samples were grown using the floating zone method. After the crystallographic axes had been aligned using a Laue camera, the single crystals were carefully cut into three small pieces with typical dimensions of 1.2 × 0.2 × 0.2 mm^3^ to have the longest directions parallel to *a*, *b,* and *c* axes. The sample size was kept nearly constant to ensure that the boundary scattering effect was similar for all samples. We note that the chemical formula for this compound should be BiCu_2_(PO_4_)O_2_, in which (PO_4_)^3−^ acts as a polyatomic ion group, while the notation in this manuscript was used to follow the former literature.

### Thermal conductivity measurements in high magnetic fields

A one-heater, two-thermometer configuration was used to measure *κ* in a Physical Property Measurement System (PPMS^TM^) for fields of up to 14 T. The calibration curves of the Cernox^TM^ thermometers at each magnetic field were measured in prior to the *κ(T*) measurement. A similar configuration with custom-made, vacuum-sealed ^3^He probe was inserted in a resistive magnet for fields of up to 35 T at the National High Magnetic Field Laboratory (NHMFL) in Tallahassee, USA. The magnetic field dependence of the Cernox^TM^ thermometers used in here has been calibrated at fixed temperatures down to 0.6 K and up to 50 T, by using a 65 T short-pulse magnet at NHMFL in Los Alamos, USA. The resistance change of the resistance thermometers due to the temperature gradient inside the sample was measured with the lock-in amplifiers (Stanford Research SR830), while applying the heat with the dc current source (Keithley 6221) and measuring the voltage difference of the heater with the nanovoltmeter (Keithley 2182). A high vacuum of well below 10^−5^ mbar was maintained throughout the measurements. For these measurements of *κ* under high magnetic fields, *H* was applied along the *a* axis.

### Kinetic theory of phonon thermal conductivity with resonant scattering

The phonon thermal conductivity of an insulating system can be explained with the equation derived from the transport theory^26^


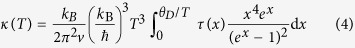


with the phonon scattering rate of


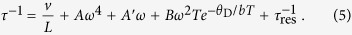


Here, *θ*_D_ is the Debye temperature of the crystal (*θ*_D_ = *v(ħ*/*k*_B_)(6*π*^2^*n*/*V*)^1/3^ where *n*/*V* is number density of atoms), *v* is averaged sound velocity of phonon, *ω* = *xk*_B_*T*/*ħ* and 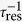 is the resonant scattering rate. *L*, *A*, *A*’, *B* and *b* are the adjustable parameters related to the strength of the phonon scattering.

Calculation based on the concept of the resonant scattering in the isolated *S* = 1/2 dimer system described above shows that the resonant scattering rate is^8^


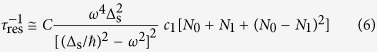


In this equation, *C* is related to the overall strength of the spin-phonon coupling, *N*_0_ is the probability of excitation process starting from one of the one-triplet, one-singlet states (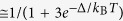), *N*_1_ is the probability of de-excitation process starting from one of the bound triplet states (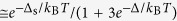) and *c*_*i*_ is the probability of the thermal excitation (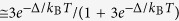). Δ_s_ is the resonance energy.

When the energy gap between the ground state and three excited states are split, the resonant scattering process for each excited configuration should be considered separately. This can be achieved phenomenologically by decomposing the Eq. (6) into three different terms.


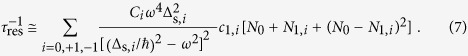


Here,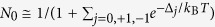, 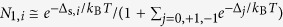 and
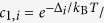
 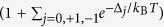. Δ_*i*_ is the energy gap between 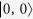 and 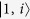 and Δ_s,*i*_ is the resonance energy of each excitation.

Although the Shastry-Sutherland lattice is considered while establishing details of this model^8^, any kind of the spin gap system can be applicable in principle since the model does not require direct information about geometry of the Shastry-Sutherland system.

### Theory of magnetic thermal conductivity

The calculation for magnetic thermal conductivity also starts from the kinetic theory of the thermal conductivity given in Eq. (1). Now we need to consider the one-dimensional dispersion of the magnetic excitations in BiCu_2_PO_6_. As we mentioned above, the energy gap between ground state (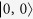) and excited states (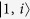) are split due to the anisotropic interactions^20^. Thus, one should rewrite the Eq. (1) for three different branches (*ε*_***k***,0_, *ε*_***k***,+1_ and *ε*_***k***,−1_).


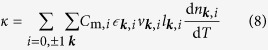


The overall constant *C*_m,*i*_ is related to the relative weight of each branch (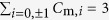). The statistical occupation for the excitation to 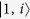 can be written as


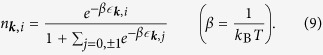


Now the formula for the magnetic thermal conductivity can be calculated as follows. For simplicity, one can assume that dispersion of the energy bands *ε*_***k***,*i*_ are same except constant shift (i.e. *ε*_***k***,1_ = *ε*_***k***,0_ + (Δ_1_ − Δ_0_) and *ε*_***k***,−1_ = *ε*_***k***,0_ + (Δ_−1_ − Δ_0_)). Then *n*_*k*__,__i_ become an explicit function of *ε*_***k***,*i*_. We also assume one-dimensional heat conduction along the *N* ladders in a unit area. By considering *v*_*k*_ = *ħ*^−1^d*ε*/d*k*, the summation over *k* can be replaced to the integration with respect to *ε*. The integral range for each excitation branch is from Δ_*i*_ (band minima) to 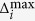 (band maxima). Further approximation such as a constant mean free path *l*_*k*_ = *l*_mag_ can be possible since the quasiparticle excitation for magnetic heat conduction dominates just close to the band minima at *k* = *k*_0_ 1. By considering all of above, the formula for magnetic thermal conductivity will be


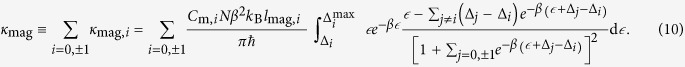


One can check that this equation reduces to


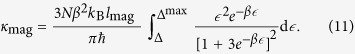


when there is no energy split among the magnetic excitations^30^.

Magnitude of the magnetic thermal conductivity is usually limited by the magnetic mean free path *l*_mag_. Various sources such as defect and spin-lattice interaction determine the magnetic mean free path. The temperature dependence of the magnetic mean free path can be given as 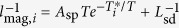, where *A*_sp_ is strength of spin-lattice interaction (interaction between phonon and magnetic quasiparticle), 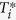 is an adjustable parameter related to the energy scale of thermal excitation and *L*_sd_ is mean distance between magnetic defects^25^,^32^. Unfortunately, this is too much simplified model to describe the magnetic mean free path, and in reality other scattering sources such as the scattering among the magnetic quasiparticles might be included as well.

## Additional Information

**How to cite this article**: Jeon, B.-G. *et al.* Giant suppression of phononic heat transport in a quantum magnet BiCu_2_PO_6_. *Sci. Rep.* **6**, 36970; doi: 10.1038/srep36970 (2016).

**Publisher’s note**: Springer Nature remains neutral with regard to jurisdictional claims in published maps and institutional affiliations.

## Figures and Tables

**Table 1:** Numerical parameters used to calculate the phonon thermal conductivity at 0 T.

	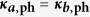
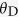 (K)	330
*L* (10^−4^ m)	3.2 ± 0.4
*L*_min_ (10^−10^ m)	6 ± 1
*A* (10^−43^ s^3^)	8 ± 1
*A*′ (10^−5^)	1.9 ± 0.1
*B* (10^−17^ s K^−1^)	1.9 ± 0.1
*b*	3.2 ± 0.3
*C*_0_ (10^53^ J^−2^ s^−1^)	<0.1
*C*_1_ (10^53^ J^−2^ s^−1^)	4.5 ± 0.6
*C*_−1_ (10^53^ J^−2^ s^−1^)	9.0 ± 1.4
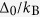 (K)	25 ± 4
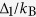 (K)	42 ± 3
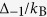 (K)	57 ± 4
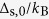 (K)	32 ± 3
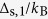 (K)	32 ± 3
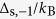 (K)	32 ± 3

*θ*_D_ = 330 K was taken from the literature^24^, and the other parameters were adjusted to reproduce the experimental data. The resonance energies for each excitation channel, Δ_s,*i*_ (*i* = 0, +1, −1), were assumed as an identical value.

**Table 2:** Δ(*H*) for calculating *κ*-*T* at various magnetic fields.

	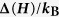 for 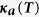	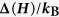 for 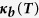
0 T	0	0
9 T		9 ± 1
14 T		18 ± 1
18 T	26 ± 1	28 ± 1
20 T	32 ± 1	33.5 ± 1

The thermal conductivity under the magnetic field can be explained by assuming the variation of the energy gaps as Δ_0_(*H*) = Δ_0_(0), Δ_+1_(*H*) = Δ_+1_(0) − Δ(*H*) and Δ_−1_(*H*) = Δ_−1_(0) + Δ(*H*), based on the magnetic field dependence of the magnetic excitation branches observed from the inelastic neutron scattering^20^.

**Table 3:** Parameters for calculating *κ*_*b*,mag_ − *T* at 0 T.

	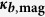
*C*_m,0_	2.3 ± 0.1
*C*_m,1_	0.14 ± 0.02
*C*_m,−1_	0.55 ± 0.04
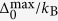 (K)	75 ± 5
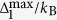 (K)	117 ± 34
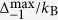 (K)	>350
*A*_sp_ (10^6^ m^−1^ K^−1^)	1.0 ± 0.1
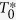 (K)	18 ± 4
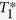 (K)	70^†^
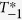 (K)	>150
*L*_sd_ (10^−7^ m)	1.0 ± 0.1

We assumed that 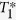 = 70 K (marked as †) based on the energy scales of 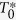 and 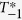, since *κ* is nearly insensitive with respect to the 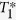 and thus it was difficult to determine its value from the fitting.
